# Setting a TRAP for phloem translational dynamics during nodulation

**DOI:** 10.1093/plphys/kiaf594

**Published:** 2025-11-15

**Authors:** Ritu Singh, Neeta Lohani

**Affiliations:** Assistant Features Editor, Plant Physiology, American Society of Plant Biologists; Department of Plant Science, University of California, Davis, CA 95616, USA; Assistant Features Editor, Plant Physiology, American Society of Plant Biologists; Department of Biotechnology, Thapar Institute for Engineering and Technology, Patiala, Punjab 147004, India

Legumes such as soybean form symbiotic partnerships with rhizobia that fix atmospheric nitrogen (N_2_) into ammonia (NH_3_), a bioavailable form that plants can assimilate, thereby reducing the need for synthetic nitrogen fertilizers. This remarkable symbiosis occurs in specialized root structures called nodules, where bacteria receive photosynthetic sugars from the plant in exchange for providing fixed nitrogen. For decades, researchers have investigated how root cells recognize compatible rhizobia, allow their entry, and establish the nodule structures that sustain this symbiosis (Zipfel and Oldroyd 2017). However, nodule formation is not solely controlled at the root level. Communication between shoots and roots ensures that the number of nodules formed matches the plant's nutritional and metabolic needs (Ferguson et al. 2019). These long-distance regulatory signals move through the vascular system, particularly the phloem, which typically transports sugars, peptides, and hormones. Although the phloem is known to deliver systemic cues that influence nodulation, a fundamental question remains: Is the phloem merely a passive transport channel, or does it actively perceive, interpret, and modulate these signals?

In a recently published *Plant Physiology* issue, Song and colleagues (Song et al. 2025) explored this by developing a phloem-specific translating ribosome affinity purification sequencing (TRAP-seq) system for soybean (*Glycine max*). TRAP-seq captures mRNAs that are being translated into proteins by tagging a ribosomal protein so that ribosome-mRNA complexes can be pulled down and sequenced (Zanetti et al. 2005). As ribosomes are ubiquitous, achieving phloem specificity required the expression of a tagged ribosomal protein exclusively in phloem cells. For this, the authors identified phloem-specific promoter *Glyma.01G040700* through laser microdissection coupled with RNA-seq and validated its specificity through published single-cell RNA-seq data (Cervantes-Pérez et al. 2024). However, the promoter's native activity was too weak for efficient TRAP capture. To amplify expression while maintaining phloem specificity, the authors employed the GAL4-UAS system in which the phloem promoter drives GAL4-VP16, which then strongly activates transcription of the tagged ribosomal protein gene. This 2-step amplification achieved high sensitivity while preserving phloem specificity, as confirmed through GUS staining and immunoblot analysis.

Using this optimized system, the authors profiled phloem translatomes at 72 h post-inoculation (hpi), representing early rhizobia infection, and 21 days post-inoculation (dpi), representing mature nodules. More than 2,636 genes were differentially translated at the early stage and over 8,422 at the late stage. Early-stage translatomes were enriched for genes involved in structural defense, hormone signaling, and nutrient mobilization, reflecting the establishment of a symplastic communication network and metabolic adaptation to rhizobial entry. By contrast, the late-stage responses shifted to solute transport, vascular differentiation, and cell wall remodeling, consistent with enhanced nutrient flux into mature nodules. Simultaneously, pathways related to cell proliferation were downregulated, indicating a developmental transition from nodule initiation to maintenance of symbiosis.

Investigation of these temporal shifts revealed that the early infection stage showed induction of Ethylene Response Factor (ERF), MYB, NAC, and C2H2 transcription factor (TF) families. Additionally, key immune regulators genes such as the receptor-like kinase *GmBAK1*, WRKY TFs, and calcium-dependent protein kinases were induced at 72 hpi but repressed or stabilized by 21 dpi. This transient activation suggests the phloem maintains immune surveillance during early infection to protect vascular tissues, then relaxes defenses once symbiosis is established. Later, during nodule maturation, Auxin Response Factors were downregulated, suggesting a suppression of auxin signaling consistent with systemic feedback processes like the autoregulation of nodulation that prevents excessive nodule formation.

Among the most intriguing findings were genes for *pectin methylesterases* (*PMEs*) and their inhibitors (*PMEIs*), which regulate plasmodesmatal permeability by influencing callose turnover (Yadav and Chattopadhyay 2014). Distinct temporal clusters of *PME* and *PMEI* transcripts indicated coordinated regulation of symplastic connectivity. Functional validation revealed that phloem-specific expression of PME domains promoted nodule formation, whereas *PMEI* overexpression reduced nodule number. These phenotypes inversely correlated with callose deposition, suggesting that *PME-PMEI* balance determines plasmodesmatal aperture and intercellular signaling capacity.

The authors also identified phloem-enriched TF *GmbHLH121*, TF that negatively regulates nodulation. RNAi knockdown of *GmbHLH121* showed increased nodule numbers without affecting classical nodulation genes like *NIN*, *NSP*, or *ENOD40*. This suggests *GmbHLH121* functions independently of canonical pathways, potentially integrating phloem-derived nutrient or stress cues (Figure 1). This expands the repertoire of known nodulation regulators.

**Figure:**
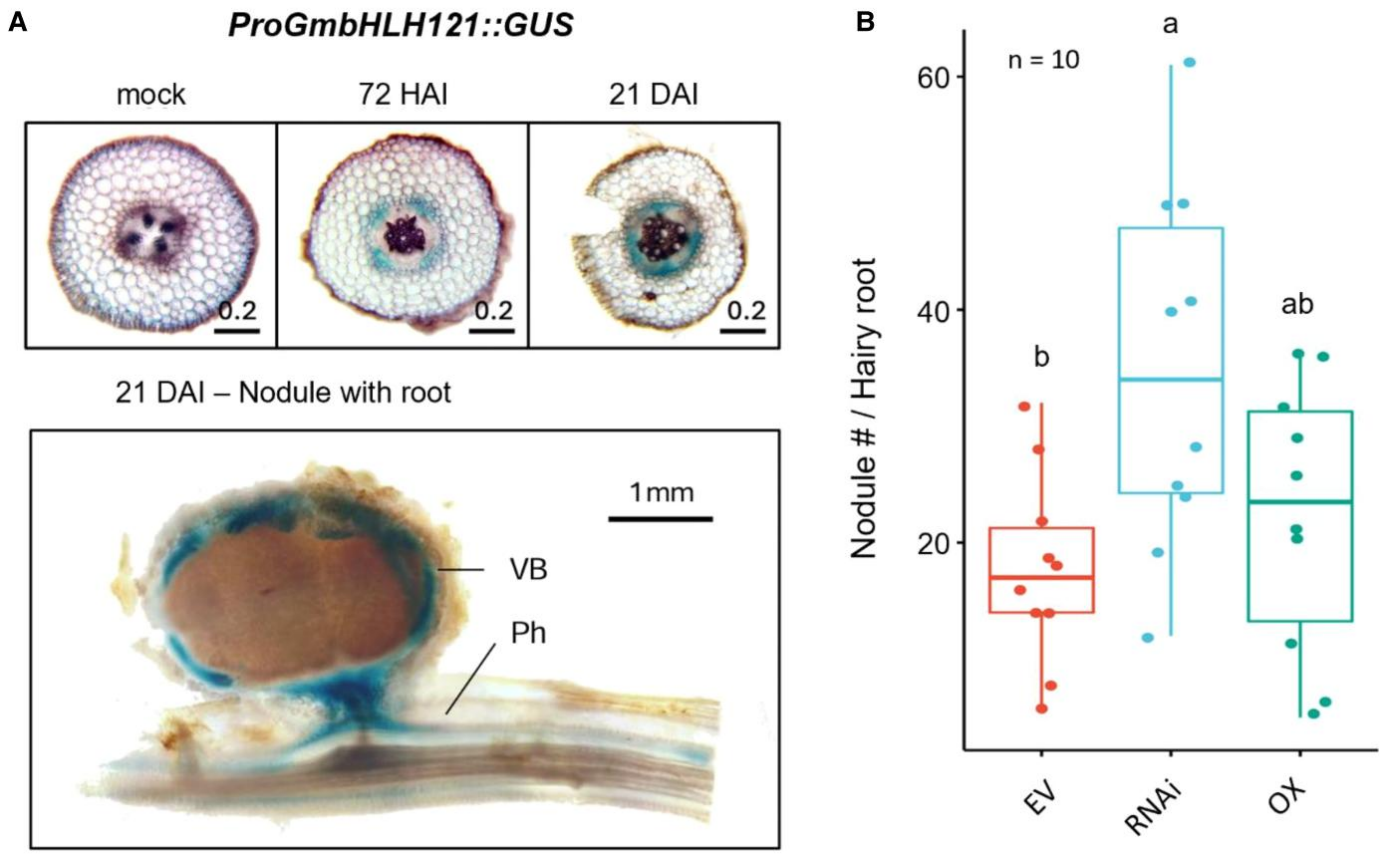
Phloem-specific TRAP-seq identifies *GmbHLH121* as a regulator of nodulation (adapted from Song et al. (2025)). **A)** Phloem-specific expression of *GmbHLH121*. Top: GUS staining in root cross-sections at mock, 72 hpi, and 21 dpi. Bottom: Mature nodule showing expression in vascular bundle (VB) and phloem (Ph). **B)**  *GmbHLH121* negatively regulates nodulation. RNAi knockdown significantly increases nodule numbers compared to empty vector (EV) control, while overexpression (OX) lines show an intermediate phenotype. Different letters indicate statistically significant differences (*P* < 0.05).

Together, these results redefined the phloem's role in legume symbiosis, revealing it as an active regulatory hub rather than a passive transport conduit. The phloem senses infection, modulates immunity, gates signal transmission through plasmodesmatal control, and coordinates nutrient allocation through translational reprogramming.

Beyond its biological insights, the GAL4-UAS–enhanced TRAP-seq approach offers a powerful, generalizable framework for cell type–specific profiling with high sensitivity and specificity, where weak promoters have previously limited progress. This system can be extended to study other contexts: Do similar phloem programs operate in other legumes, during mycorrhizal symbioses, or in stress responses? From an applied perspective, targeting phloem-specific regulators like modulating *PME-PMEI* balance, manipulating *GmbHLH121* TF, or engineering phloem transporter could provide systemic control over symbiotic efficiency. As agriculture seeks sustainable alternatives to synthetic nitrogen fertilizers, understanding phloem-mediated regulation of biological nitrogen fixation offers promising avenues for improving legume performance and nutrient use efficiency in crop systems.

## Recent related articles in *Plant Physiology*:

- Xiao et al. (2025) described how local, stage-specific auxin biosynthesis and transport are indispensable for nodule organogenesis in *Medicago truncatula*.
- Kawade et al. (2024) described how *Lotus japonicus* plants use systemic controls like the autoregulation of nodulation to limit root nodule formation and manage their carbon investment in symbiosis.

## Data Availability

No data is generated in this study.
